# Supplementary material for: RNA Biomarkers as a Response Measure for Survival in Patients with Metastatic Castration-Resistant Prostate Cancer
Source: Cancers (Basel). 2021 Dec 14;13(24):6279. doi: 10.3390/cancers13246279 (PMC8699291; doi:10.3390/cancers13246279)
Supplement: Supplementary file 1 [file cancers-13-06279-s001.zip › cancers-1430276-supplementary.pdf]

cancers

MDPI

Supplementary Materials: RNA Biomarkers as a Response Measure for Survival in Patients with Metastatic Castration-Resistant Prostate Cancer

Emmy Boerrigter, Guillemette E. Benoist, Inge M. van Oort, Gerald W. Verhaegh, Anton F.J. de Haan, Onno van Hooij, Levi Groen, Frank Smit, Irma M. Oving, Pieter de Mol, Tineke J. Smilde, Diederik M. Somford, Paul Hamberg, Vincent O. Dezentjé, Niven Mehra, Nielka P. van Erp and Jack A. Schalken

1. Results

1.1. Biomarker Levels in Healthy Volunteers

The biomarkers were measured in 30 healthy volunteers (10 men <35 year, 10 men between 55–70 year, and 10 women (no age restriction)). Levels of *NAALADL2-AS2* were only measured in 10 age-matched men. Levels of KLK3 mRNA were not measured in healthy controls, since previous work showed that KLK3 is only present in patients with prostate cancer. The average Cp values in healthy controls are shown in Table S1 and were used as a reference.

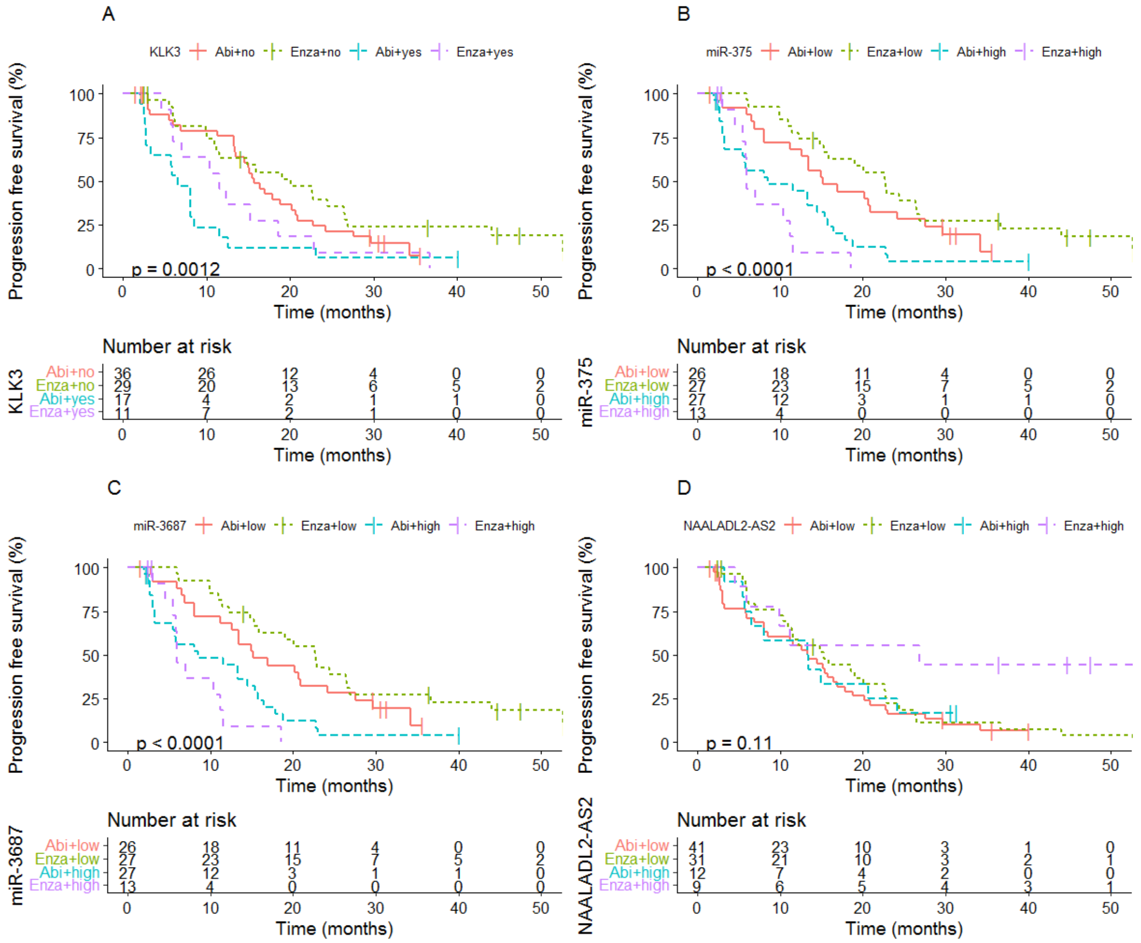

Figure S1. Kaplan-Meier analysis of progression-free survival based on baseline values, divided per treatment (Abi = abiraterone acetate and Enza = enzalutamide), for (**A**). *KLK3*, (**B**). miR-375, (**C**). miR-3687 and (**D**). *NAALADL2-AS2*.

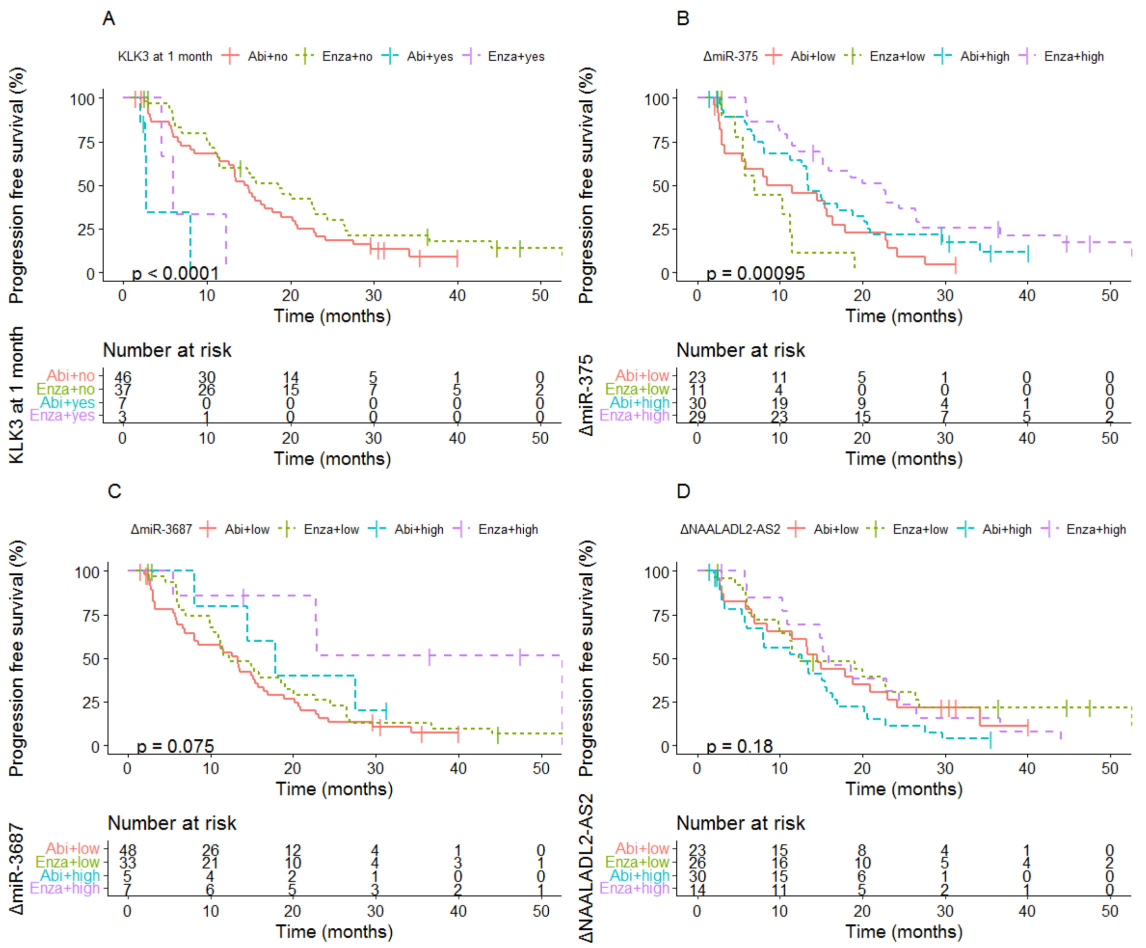

Figure S2. Kaplan-Meier analysis of progression-free survival based on delta values at 1 month, divided per treatment (Abi = abiraterone acetate and Enza = enzalutamide), for (**A**). *KLK3*, (**B**). miR-375, (**C**). miR-3687 and (**D**). *NAALADL2-AS2*.

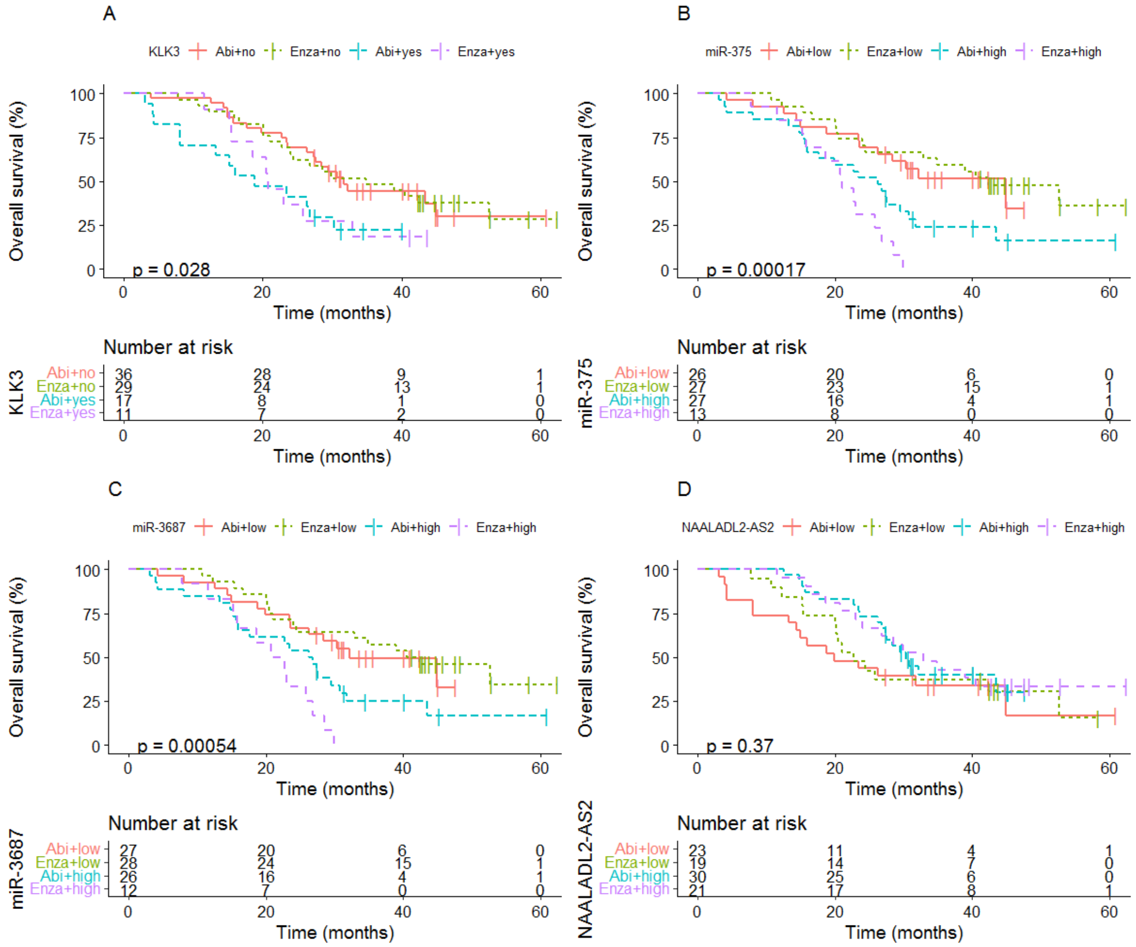

Figure S3. Kaplan-Meier analysis of overall survival based on baseline values, divided per treatment (Abi = abiraterone acetate and Enza = enzalutamide), for (**A**). *KLK3*, (**B**). miR-375, (**C**). miR-3687 and (**D**). *NAALADL2-AS2*.

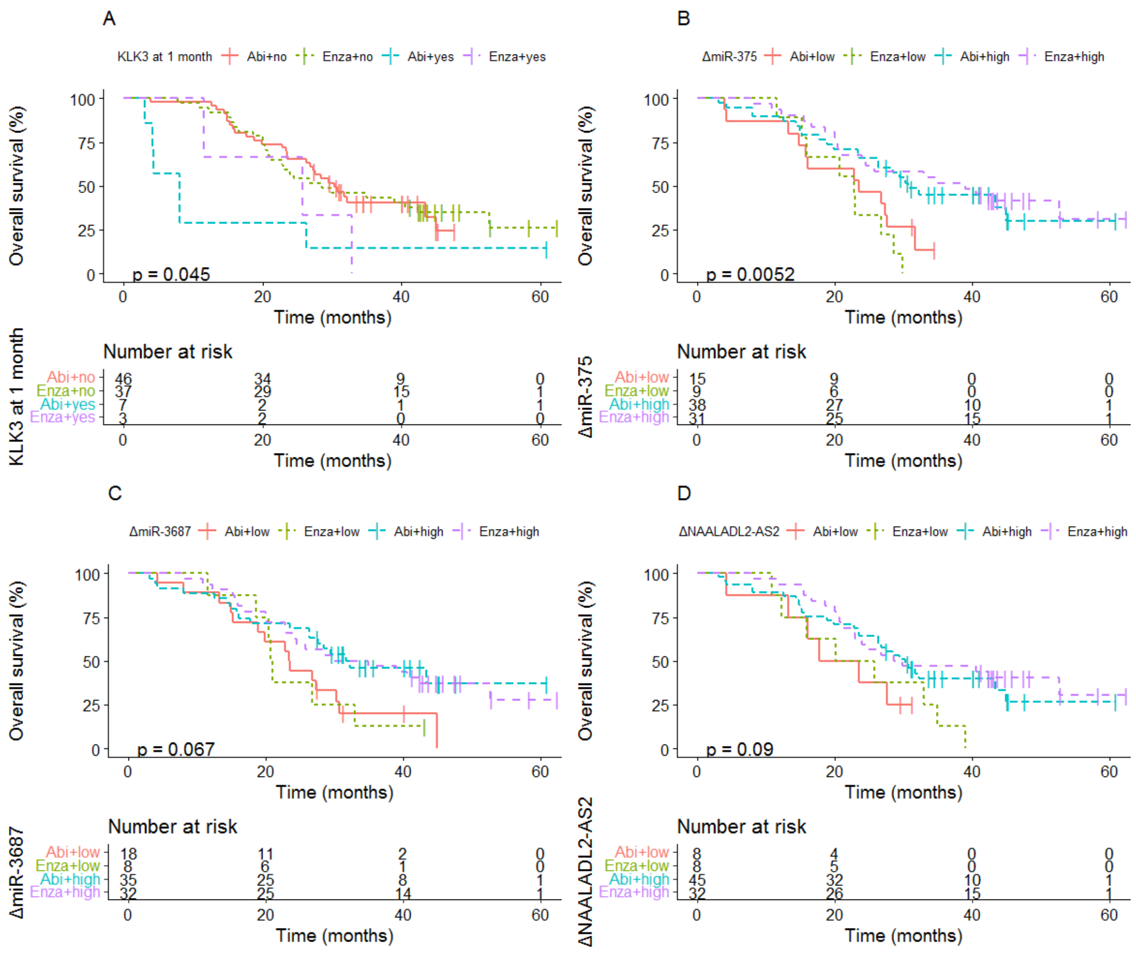

Figure S4. Kaplan-Meier analysis of overall survival based on delta values at 1 month, divided per treatment (Abi = abiraterone acetate and Enza = enzalutamide), for (**A**). *KLK3*, (**B**). miR-375, (**C**). miR-3687 and (**D**). *NAALADL2-AS2*.

Table S1. Overview of biomarker expression levels in healthy controls.

	Biomarkers (Cp-Values)		
	**miR-375**	**miR-3687**	***NAALADL2-AS2***
Men (≤35 y/o)	31.03	32.06	-
Men (≤35 y/o)	31.48	30.97	-
Men (≤35 y/o)	31.08	32.07	-
Men (≤35 y/o)	31.14	31.30	-
Men (≤35 y/o)	31.24	31.61	-
Men (≤35 y/o)	31.85	32.24	-
Men (≤35 y/o)	30.73	31.06	-
Men (≤35 y/o)	31.68	31.86	-
Men (≤35 y/o)	31.34	32.20	-
Men (≤35 y/o)	30.66	31.79	-
Men (53-70 y/o)	30.80	31.89	35.59
Men (53-70 y/o)	32.30	31.88	32.06
Men (53-70 y/o)	31.86	31.56	35.27
Men (53-70 y/o)	31.46	31.02	35.12
Men (53-70 y/o)	31.29	31.01	32.37
Men (53-70 y/o)	32.47	31.20	32.88
Men (53-70 y/o)	31.50	31.54	34.38
Men (53-70 y/o)	31.90	31.05	33.84

Men (53-70 y/o)	32.74	30.57	32.03
Men (53-70 y/o)	32.21	30.68	33.18
Female	30.77	28.03	-
Female	30.68	31.21	-
Female	29.99	30.83	-
Female	31.68	31.43	-
Female	31.57	31.74	-
Female	30.82	31.07	-
Female	29.71	30.84	-
Female	30.95	31.32	-
Female	31.43	31.18	-
Female	31.03	31.11	-

1.2. Cut-Off Values Used for Kaplan-Meier Analysis of the Biomarkers

The cutoff values for miR-375, miR-3687 and *NAALADL2-AS2* at baseline (relative to healthy controls) and for the delta values at 1 month, calculated with maximally selected rank statistics, are shown in table 1. Since *KLK3* was only detectable in a subset of patients, *KLK3* values were dichotomized, i.e. detectable yes or no. The cutoff value used for *KLK3* at 1 month was detectable yes or no after 1 month of therapy.

Table S2. Cut-off values used for Kaplan-Meier analysis of the biomarkers.

	**miR-375**	**miR-3687**	***NAALADL2-AS2***
Cutoff PFS baseline	2.08	0.76	2.03
Cutoff PFS delta at 1 month	−0.41	1.39	0.097
Cutoff OS baseline	2.08	2.21	2.52
Cutoff OS delta at 1 month	−0.65	-0.72	−0.77
